# Global–local consistency benefits memory‐guided tracking of a moving target

**DOI:** 10.1002/brb3.2444

**Published:** 2021-12-03

**Authors:** Tingting Chen, Jinhong Ding, Guang H. Yue, Haoqiang Liu, Jie Li, Changhao Jiang

**Affiliations:** ^1^ School of Education Beijing Dance Academy Beijing P.R. China; ^2^ Beijing Key Laboratory of Learning and Cognition & School of Psychology Capital Normal University Beijing P.R. China; ^3^ Human Performance and Engineering Research, Kessler Foundation West Orange New Jersey; ^4^ School of Education Shangdong Woman University Jinan P.R. China; ^5^ Institute of Psychological Sciences Hangzhou Normal University Hangzhou P.R. China; ^6^ Beijing Key Lab of Physical Fitness Evaluation and Tech Analysis Capital University of Physical Education and Sports Beijing P.R. China

**Keywords:** eye velocity trace, global–local consistency, prediction motion tasks, velocity gain, VSTM

## Abstract

**Introduction:**

Previous findings have demonstrated that several Gestalt principles do facilitate VSTM performance in change detection tasks. However, few studies have investigated the role of and time‐course of global–local consistency in motion perception.

**Methods:**

Participants were required to track a moving target surrounded by three different backgrounds: blank, inconsistent, or consistent. Global–local objects were be bound to move together (covariation). During the PMT, participants had to follow the moving target with their eyes and react as fast as possible when the target had just vanished behind the obstruction or would arrive at a predetermined point of interception. Variable error (VE) and constant error (CE) of estimated time‐to‐contact (TTC) and gain of smooth pursuit eye movements were calculated in various conditions and analyzed qualitatively.

**Results:**

Experiment 1 established the basic finding that VSTM performance could benefit from global–local consistency. Experiment 2 extended this finding by eye‐tracking device. Both in visible phase and in occluded phase, CEs were smaller for the target in a consistent background than for the target in an inconsistent background and for the target in a blank background, with both differences significant (*p*s < .05). However, the difference in VE among three conditions was not significant. At early stage (100–250 ms), later stage (2750–3000 ms), and termination stage (5750–6000 ms) of smooth pursuit, the velocity gains were higher in the trials with consistent backgrounds than in the trials with inconsistent backgrounds and blank backgrounds (*p*s < .001). With the exception of 100–250 ms phase, the means did not differ between the inconsistent background and the blank background trials (*p*s > .1).

**Conclusions:**

Global–local consistency could be activated within the first few hundred milliseconds to prioritize the deployment of attention and eye movement to component target. Meanwhile, it also removes ambiguity from motion tracking and TTC estimation under some unpredictable conditions, leading to the consistency advantage during smooth‐pursuit termination phase. Global–local consistency may act as an important information source to TTC estimation and oculomotor response in PMT.

## 1 INTRODUCTION

When people observe visual scenes, an important fundamental question is how the visual system organizes the incoming stream of visual information. Early Gestalt theorists have formulated a number of principles that aim to capture the regularities according to which perceptual input is organized or grouped into meaningful units or Gestalts (Koffka, 1922; Wagemans et al., 2012). For example, the principle of proximity refers to parts of the visual field that are close to each other tend to be grouped into one whole (which could be a pattern, a texture, or an object) whereas the principle of similarity states that elements will tend to be grouped together if their attributes are perceived as related (e.g., in color or shape). The Gestalt principle of grouping by good continuation states that we tend to group lines or curves that follow an established direction.

Since the early works on the Gestalt theory of scene perception, a considerable amount of research has been conducted on the global–local interaction. A seminal work by Navon (1977) has demonstrated that the global precedence effect is a prevailing property of object‐background processing. Navon presented compound letters representing larger figures (global configurations), which were spatially constructed from a suitable arrangement of smaller figures (local elements), and observed an advantage in the processing of global configurations over local elements (i.e., faster judgments of local shape when local and global shapes are consistent, but not vice versa). Critically, when global configurations and local elements were inconsistent, responses to the local elements were subject to interference from the global configurations, but local features did not interfere with global perception, which was termed as the “global interference effect.” Further investigation has shown, whether compound letters or compound figures, inconsistent stimuli were responded to more slowly than both consistent and neutral stimuli, which did not differ from each other (Poirel et al., 2008). Evidences from event‐related brain potential (ERP) studies have shown that consistent stimuli elicited larger N1 amplitude (150–220 ms), which occurs at the early steps of visual processing (Beaucousin et al., 2013).

The global–local consistency effects examined in form perception seem to be generalized to motion perception. In early studies, the Gestalt principle of grouping by common fate indicates that an invisible form composed of randomly arranged dots against a dotted background becomes immediately visible as soon as it moves, by virtue of the common fate of its dots, which all move together with a common speed and direction. The spatial integration of target and background motion signals has been studied by having observers track a pursuit target in the presence of a second moving object, or in front of a stationary or moving textured background (Spering & Gegenfurtner, 2008). Generally, the pursuit of a moving object on a stationary textured background was hampered, and initial acceleration and steady‐state velocity were lowered (Masson et al., 1995; Spering & Gegenfurtner, 2008). Nevertheless, the results are more complicated when tracking a moving target on a moving background. A background moving in the same direction as the pursuit target raised pursuit velocity, while a background moving in the reverse direction lowered eye velocity (Masson et al., 1995). Pursuit was not impacted by the alterations in background velocity when the background moved in opposite direction of the pursuit target (Spering et al., 2006; 2007; 2008). Therefore, motion signals (e.g., direction consistency, velocity consistency) from the local target and global background have to be integrated hierarchically in order to extract a precise velocity signal for initiating and maintaining an accurate eye movement (Eggert et al., 2009; Ladda et al., 2007; Ogawa et al., 2009; Spering & Gegenfurtner, 2008).

Throughout visual tracking of a moving target, what often happens is that a moving target is momentarily obstructed by additional objects and vanished from view (Albright & Stoner, 2002). In this case, visual short‐term memory (VSTM) allows us to temporarily store and process relevant information from the visual world across saccades and other visual interruptions. VSTM is defined as short‐term memory for nonverbal, visual information, a buffer that temporarily stores visual information before it can be further processed. Previous findings have demonstrated that several Gestalt principles (e.g., connectedness, common region, and spatial proximity) do facilitate VSTM performance in change detection tasks (Peterson & Berryhill, 2013; Woodman et al., 2003; Xu, 2006; Xu & Chun, 2006). However, relatively little is known about the role that global–local consistency play in visual short‐term memory storage. In other words, can global–local consistency be an approach to enhance VSTM function by optimizing the processing of information? In the present study, prediction motion task (PMT) is designed to investigate the observers’ estimate ability of the precise position of a moving object while lacking visual information input. In a typical PMT, an independent target moved at constant velocity along the frontoparallel plane and then vanished behind the obstruction; the participants were asked to press a button when they thought the object would arrive and touch a predetermined point of interception (Benguigui & Bennett, 2010; Bennett & Benguigui, 2016; Bennett et al., 2010; DeLucia et al., 1998; Flavell et al., 2018; Makin & Bertamini, 2014; Makin & Chauhan, 2014; Makin & Poliakoff, 2011; Makin et al., 2008; 2009; Vicovaro et al., 2019). In such situations, how is the global–local consistency utilized by observers to estimate the precise location of an occluded target?

To investigate the time‐course of global–local consistency on motion perception, we asked participants to track a moving target surrounded by three different backgrounds: consistent background, inconsistent background or blank background. Global–local objects were be bound to move together (covariation). Each condition was repeated a few times in random order to construct a representative estimation. During the PMT, participants had to follow the moving target with their eyes and react as fast as possible when the target had just vanished behind the obstruction or would arrive at a predetermined point of interception. Eye movements were recorded in various conditions and analyzed qualitatively to ensure that the participants acted as directed. We hypothesized that the consistent background eased perceptual encoding at the initial stages of visual processing of an object, and also modulated information processing in VSTM. Thus, global–local consistency effects are expected to be significant during visually guided tracking as well as during memory‐guided tracking.

## 2 EXPERIMENT 1

### 2.1 Methods

#### 2.1.1 Participants

G*power is a free software that helps researchers to calculate the sample size needed when conducting an experiment. We set the power value 1 − *β* = 0.80 and the effect size *f* ^2^ = 0.25, which is a medium effect size value (Cohen, 1992) and got the estimated total sample size to be 19. A group of 25 right‐handed undergraduate or graduate students at the Capital University of Physical Education and Sports took part in the experiment for cash compensation. They were between 18 and 26 years of age and reported having normal or corrected‐to‐normal visual acuity and normal color vision.

#### 2.1.2 Design and stimuli

A 2 × 3 × 2 within‐subjects factorial design was used for the experiment 1, with the first factor referring to the target visibility (visible or occluded), the second to the local target (triangle or circle), and the third to the global–local consistency (blank, inconsistent, or consistent). The visual display and response system were controlled from a computer running E‐prime scripts (Psychology Software Tools, Pittsburgh, PA).

**FIGURE 1:**
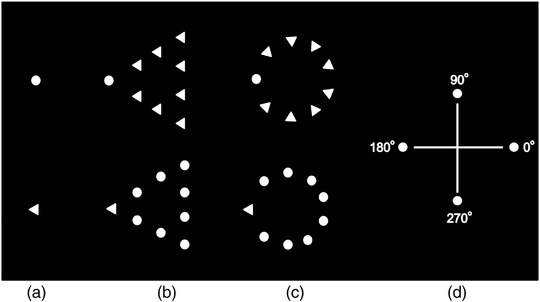
Global–local conditions employed in the experiment 1. The white filled circle or triangle to be tracked was embedded in one of the three backgrounds: blank, inconsistent, or consistent. The target circle was randomly presented at the four orientations on the screen

Before implementing the experiment, we recruited 70 undergraduate or graduate students to rank the candidate stimuli with which stimulus is more likely to occur in natural world. Chi‐square test and pairwise comparisons provide the support for screening the global background conditions (χ^2 ^= 9.886, *p *= .02). After initial evaluation, there were three types of backgrounds: (i) the blank condition only displayed a small target circle or a small target triangle, without any background elements; (ii) the consistent condition presented a big triangular shape which consisted of a target triangle and eight small circles that were arranged in a triangular pattern, or a big circle shape which consisted of a target circle and eight small triangles that were arranged in a circular pattern; (iii) the inconsistent condition presented a big triangular shape which consisted of a target circle and eight small triangles that were arranged in a triangular pattern, or a big circle shape, which consisted of a target triangle and eight small circles that were arranged in a circular pattern. The triangular background elements were all apex pointing toward the same direction (approximately 2.43° × 3.81°), while the circular background elements all had their apex pointing toward the center of the circle (3.82° diameter) (see Figure 1).

As depicted in Figure 1, the target was a white filled triangle or circle (0.38° in diameter), which was randomly presented at one of the four selected orientations on the left side of the screen (see Figure 1d). The target and the background elements were rotating together in a uniform circular motion while moving toward the occlusion zone, just like an imaginary rolling wheel from left to right across the screen. Once the target of visually tracking reached the occlusion zone, all of these elements disappeared one by one behind the occlusion zone. Participants had to track the rolling path of the target with their eyes as accurately as possible and react as fast as possible when the target had just vanished behind the obstruction or would arrive at a predetermined point of interception.

#### 2.1.3 Behavioral procedures

**FIGURE 2:**
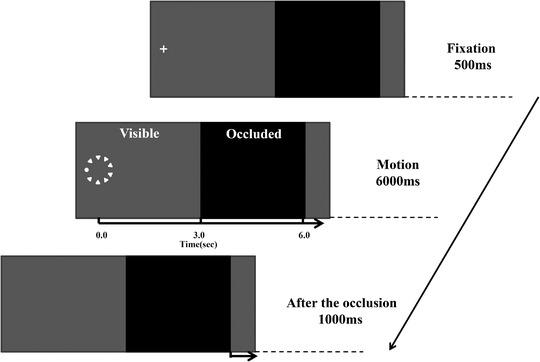
Sequence of events in a trial

Each trial lasted for 7500 ms. At the beginning of each trial (Figure 2), a white fixation point (a white + sign, 0.2° in visual angle) was presented on the left side of the screen as a trial‐start cue. After 500 ms, the fixation point was substituted by any of six display stimuli, with the target (in the consistent or the inconsistent background) moved from left to right across the screen in the gray area. The observers initially focused on the fixation point and then followed the white filled target with their eyes, tracking its motion for 6000 ms. After 3000 ms, the target moved behind the dark gray occluding bar (see Figure 2). We told the participants that the target continuously moved at the same velocity beyond the occluded bar. The participants had to push the left mouse button to mark when the target went into the left side of the dark gray occlusion bar and they had to push the button a second time when they thought that the target would arrive at the right side of the occlusion bar. However, following the occlusion period, the target did not appear again in reality. Since our preliminary results showed that the stimuli reappearing after the occlusion result in the ceiling effect of observers predicting, and likewise for when the target presented on the fixed orientation. Therefore, the target in our experiment randomly appears at one of the four selected orientations and never reappear after occlusion to avoid this ceiling effect. There was a 1000‐ms waiting period before the initiation of a new trial. We asked the participants to push the button as fast and accurately as they could. Before the experiment, all participants finished 12 practice trials to acquaint them with the task and the stimuli.

There were ten blocks of 48 trials (total 480 trials). Each block includes all six conditions. All trials were randomized in each block. The target was presented at four possible starting locations (see Figure 1d) with equal probability. There was a 1‐min break between the blocks. Instructions were given to the subjects at the start of the experiment.

### 2.2 Results and discussion

As a perceptual measure, we recorded the actual times when the target disappeared at the left border of the occlude and the estimated times when the participant pressed the left mouse button to indicate that the target had reached the left border of the occlude. Similarly, we also recorded the actual times when the target arrived at the right border of the occlude and the estimated times when the participant pressed the left mouse button again to indicate that they thought the occluded process was completed. In both the visible phase and the occluded phase, constant error (CE) of estimated time‐to‐contact (TTC) during PMT was calculated for each trial and corresponded to the signed difference between the actual arrival time and the time estimated by the subject. Trials with values more than three standard deviations above or below the individual mean were excluded before computing the overall mean and standard deviation. Furthermore, we computed the variable error (VE; the standard deviation of errors in different conditions) (Benguigui & Bennett, 2010; Bennett & Benguigui, 2016; Flavell et al., 2018; Makin & Bertamini, 2014; Makin & Chauhan, 2014; Vicovaro et al., 2019).

Mean and standard deviation measures of CE under different conditions are presented in Table 1. Results of repeated ANOVA of 2 (visible or occluded) × 3 (blank, inconsistent, or consistent) × 2 (triangle or circle) for CE showed the following main effects to be significant: target visibility, *F* (1, 24) = 213.00, *p *< .001, *η*_p_^2^ = .899, and target‐background consistency, *F* (2, 48) = 7.67, *p *< .005, *η*_p_^2^ = .24. The interaction of target visibility and consistency was also significant, *F* (2, 48) = 4.73, *p *< .05, *η*_p_^2^ = .165. No other effects or interactions reached significance (see Table 2).

**TABLE 1:** Means and standard deviations for CE under different conditions (*M* ± *SD*)

	Visible phase		Occluded phase	
	A target circle	A target triangle	A target circle	A target triangle
**Blank condition**	123.87 ± 12.68	132.82 ± 12.69	437.53 ± 35.72	460.55 ± 33.22
**Inconsistent condition**	108.64 ± 8.15	136.17 ± 14.72	421.89 ± 36.92	475.69 ± 53.31
**Consistent condition**	101.89 ± 8.93	114.38 ± 21.01	312.41 ± 37.45	311.78 ± 47.46

Table 3 shows mean and standard deviation measures of VE under different conditions. A 2 (visible or occluded) × 3 (blank, inconsistent, or consistent) × 2 (triangle or circle) repeated ANOVA was conducted on VE. Table 4 shows the ANOVA summary table for all the dependent variables. The main effect of both target visibility and consistency was significant, *F* (1, 24) = 184.30, *p *< .001, *η*_p_^2^ = .885, *F* (2, 48) = 6.40, *p *< .005, *η*_p_^2^ = .211, and the target visibility × consistency interaction was also significant, *F* (2, 48) = 4.68, *p *< .05, *η*_p_^2^ = .163. No other effects or interactions reached significance.

**TABLE 2:** Results of repeated ANOVA of 2 (visible or occluded) × 3 (blank, inconsistent, or consistent) × 2 (triangle or circle) for CE

	*Df*_1_	*Df*_2_	*F*	*p*	*η_p_* ^2^
**Target visibility**	1	24	213.00	.000	.899
**Target shape**	1	24	0.98	.332	.039
**Target‐background consistency**	2	48	7.67	.003	.242
**Target visibility × consistency**	2	48	4.73	.017	.165
**Target visibility × target shape**	1	24	0.08	.786	.003
**Target shape × consistency**	2	48	0.45	.637	.018
**Target visibility × consistency× target shape**	2	48	0.14	.852	.006

**TABLE 3:** Means and standard deviations for VE under different conditions (*M* ± *SD*)

	Visible phase		Occluded phase	
	A target circle	A target triangle	A target circle	A target triangle
**Blank condition**	90.12 ± 6.12	102.85 ± 7.25	419.04 ± 66.63	406.60 ± 37.15
**Inconsistent condition**	91.13 ± 7.53	98.84 ± 10.89	395.01 ± 31.97	403.44 ± 50.59
**Consistent condition**	82.26 ± 7.75	95.95 ± 9.83	255.42 ± 39.76	313.85 ± 24.11

**TABLE 4:** Results of repeated ANOVA of 2 (visible or occluded) × 3 (blank, inconsistent, or consistent) × 2 (triangle or circle) for VE

	*Df*_1_	*Df*_2_	*F*	*p*	*η_p_* ^2^
**Target visibility**	1	24	184.30	.000	.885
**Target shape**	1	24	0.40	.534	0.016
**Target‐background consistency**	2	48	6.40	.004	.211
**Target visibility × consistency**	2	48	4.68	.016	.163
**Target visibility × target shape**	1	24	0.02	.878	.001
**Target shape × consistency**	2	48	0.43	.631	.017
**Target visibility × consistency× target shape**	2	48	0.46	.628	.019

Experiment 1 confirmed that target‐background consistency enhances VWM performance in the PMT. Observers’ behavioral response were more accurate for a moving target in the consistent background than the blank background and the inconsistent background. Moreover, facilitation of the consistent background on a moving object was found to be significant during both visually guided tracking and memory‐guided tracking, irrespective of the target shape. A possible explanation is that a target circle moving in a uniform circular motion is commonplace. Meanwhile, people can also obtain the ability of identifying and tracking a target triangle moving in a uniform circular motion by learning and training. Although no difference was found between the two target types, tracking a moving circle seems to be easier and more stable than tracking a moving triangle. Thus, we further investigated the visual characteristics of and time‐course of object‐background consistency during tracking a target circle in PMT.

## 3 EXPERIMENT 2

### 3.1 Methods

#### 3.1.1 Participants

All of the experimental procedures were approved by and conducted in accordance with the guidelines and regulations of the Department of Psychology Institutional Review Board (IRB) at Capital Normal University. Participants came from affiliates of Capital Normal University and provided informed consent in accordance with the IRB guidelines of Capital Normal University.

We set the power value 1 − *β* = 0.80 and the effect size *f* ^2^ = 0.25, which is a medium effect size value (Cohen, 1992) in the G*Power software, and got the estimated total sample size to be 28. Due to resource constraints and drop‐out rate of participants, however, we only recruited 22 right‐handed undergraduate or graduate students (50% female; mean age = 22.5 years, SD = 2.0) as subjects in this experiment and all participants received monetary compensation for it. This sample size was also similar to that used in most of the previous eye‐tracking experiments (Coppe et al., 2010; Jiang et al., 2016; Spering & Gegenfurtner, 2008). According to Daniël Lakens (https://psyarxiv.com/9d3yf/), if it is not possible to increase the sample size, the data analysis should not focus on *p* values, but on the effect size and the confidence interval, which are reported in Results of the present study to reflect the minimal statistically detectable effect. All subjects had normal or corrected‐to‐normal visual acuity with none diagnosed of cognitive or neurological disorders.

#### 3.1.2 Design and stimuli

We utilized a 2 × 3 within‐subjects factorial design, with the first factor pertaining to the target visibility (visible period or occluded period) and the second factor pertaining to the target‐background consistency (blank, inconsistent, or consistent). The experiment was performed in a dimly lit room. Subjects were seated in the middle of the room in front of a 17‐inch CRT (Cathode Ray Tube, CRT) computer screen (1024 × 768 dpi resolution) and they viewed it from 60 cm beyond the location of the eyes of the seated participant. The visual display and response system were controlled from a computer running E‐prime scripts (Psychology Software Tools, Pittsburgh, PA).

In most studies of ocular tracking or motion extrapolation, participants (humans or monkeys) were usually asked to fixate and then track a small ball (e.g., Delle Monache et al., 2019; Khoei et al., 2013) or a laser spot (Kodaka et al., 2004; Liston et al., 2014; Vercher et al., 1988; Orban de Xivry et al., 2008) or a circular dot (Barnes et al., 2000; Ding et al., 2009; Masson et al., 1995; 2001; Niemann et al., 1997; Souto et al., 2014). Visual processing of biological motion has traditionally been investigated with the point‐light walker, composed of a number of point‐light dots depicting the motions of an agent's major joints (Coppe et al., 2010; Gertz et al., 2016; Johansson, 1973; Lange & Lappe, 2006; Yu et al., 2019). These previous research and studies provide the support for us to designate a small circle as the target of ocular tracking. As depicted in the upper region of Figure 1, the target was a white filled circle (0.38° in diameter), which was randomly presented at one of the four selected orientations on the left side of the screen (see Figure 1d).

All of the display elements were of approximately the same size. The target circle measured 0.38° in diameter. Each context triangle subtended approximately 0.44° (horizontally) × 0.38° (vertically) of visual angle, with equal distances between adjacent triangles. Stimuli were presented in white on a homogeneous gray context with a dark gray occlusion bar on the right part of the screen (Figure 2). The display area (the visible area plus the occluded area) subtended 25.99° (horizontally) × 19.64° (vertically) in a visual angle. Across conditions, the linear velocity of the uniform circular motion was approximately 10 °/s and the rotational speed of the uniform circular motion was approximately 298.8 °/s.

#### 3.1.3 Oculomotor recording and data analysis

Experimental protocols followed Experiment 1. There were five blocks of 60 trials (total 300 trials). Participants was required to follow the white filled target circle in three different conditions with their eyes. We discarded any data more than three standard deviations away from the mean during each experimental condition (0.7% of responses in total) and then we computed CE and VE.

The participant's eye position was recorded with the SMI iView X RED Remote Eye‐tracking Device, which is a remote tracking system that computes the gaze utilizing the reflection of a near infrared light from the cornea and pupil of one eye with a sampling rate of 250 Hz. Nine‐point calibrations were conducted at the start of every block. The eye‐tracking computer was synchronized to the E‐prime computer via a parallel port cable. The eye movement data were scored offline. Blinks, drifts, and other artifacts were identified and removed from the oculomotor data (3.1% of the responses in all). Saccades were first identified as points in the acceleration trace exceeding a threshold (±2.5 SD of baseline noise: ≈750−1500°/s^2^). On the rare occasions when the use of the acceleration threshold failed to identify a saccade, both a velocity threshold (>40°/s) and the amplitude range (0.3° to 5°) were applied (Bennett & Barnes, 2003). Those saccades were removed from the smooth eye velocity trace. The smooth pursuit gains were calculated for each trial as the ratio of the average eye velocity to the target velocity.

### 3.2 Results

#### 3.2.1 Behavioral results

**FIGURE 3:**
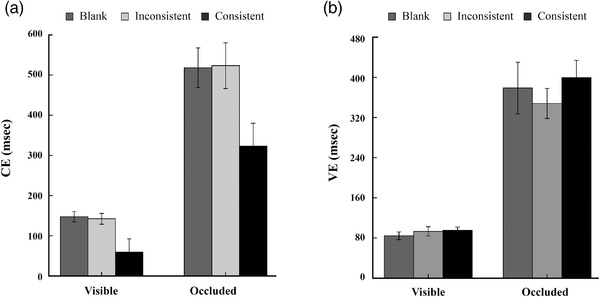
Constant error and variable error for each condition. Constant error (CE) corresponds to the signed difference between the actual arrival time and the time estimated by the subject. Variable error (VE) was defined by the standard deviation of errors in different conditions

For CE (see Figure 3a), a 2 (target visibility: visible vs. occluded) × 3 (global–local consistency: blank, inconsistent, consistent) repeated measures ANOVA were conducted. Results showed a significant main effect of target visibility, *F* (1, 21) = 49.35, 95% CI 238.50–439.07, *p *< .001, *η*_p_^2^ = .70, with CE larger in memory guided tracking (*M* = 455 ms, *SD* = 50.2) than in visually guided tracking trials (*M* = 116 ms, *SD* = 15.8). The main effect of consistency was also significant, *F* (2, 42) = 26.35, *p* < .001, *η*_p_^2^ = .56. Observers were more accurate tracking the target in consistent backgrounds (*M* = 191 ms, *SD* = 34.9) than in inconsistent backgrounds (*M* = 333 ms, *SD* = 30.9) (95% CI 77.95–206.17, *p* < .001) or blank backgrounds (*M* = 332 ms, *SD* = 27.5) (95% CI 73.10–210.81, *p* < .001). Importantly, the interaction of target visibility and consistency was also significant, *F* (2, 42) = 4.03, *p* < .05, *η*_p_^2^ = .16, suggesting that target visibility affected the magnitude of the consistency effect.

To examine these interactions, we analyzed the target‐visible and ‐occluded CE in separate ANOVAs. When the targets were visible, the main effect of consistency was significant, *F* (2, 42) = 7.04, *p *< .005, *η*_p_^2^ = .25. Pairwise comparisons using the Bonferroni correction further showed that CE were significantly smaller for the target in a consistent background (*M* = 59 ms, *SD* = 33.3) than for the target in an inconsistent background (*M* = 142 ms, *SD* = 13.4) (95% CI 1.61–164.87, *p* < .05) and for the target in a blank background (*M* = 147 ms, *SD* = 12.7) (95% CI 2.81–174.15, *p* < .05). However, the latter two conditions did not differ from each other, *p* > .1. Similarly, when the targets were occluded, a significant difference also occurred among three backgrounds in CE, *F* (2, 42) = 19.13, *p* < .001, *η*_p_^2^ = .48. That is, CE was also smaller for the target in a consistent background (*M* = 323 ms, *SD* = 57.3) than for the target in an inconsistent background (*M* = 524 ms, *SD* = 56.8) (95% CI 89.95–311.78, *p* < .001) and for the target in a blank background (*M* = 518 ms, *SD* = 49.2) (95% CI 96.68–294.18, *p* < .001). CE did not differ between the inconsistent background and blank background trials, *p* > .1. Thus, the consistency effect was found not only in visually guided tracking, but also in memory‐guided tracking.

Analysis of VE (see Figure 3b) indicated a main effect of target visibility, *F* (1, 21) = 116.22, 95% CI 229.59–339.35, *p* < .001, *η*_p_^2^ = .85, with a significant difference between the memory guided tracking (*M* = 375 ms, *SD* = 26.5) and the visually guided tracking trials (*M* = 91 ms, *SD* = 6.3). There was no main effect of consistency, *F* (2, 42) = 0.55, *p* = .52, *η*_p_^2^ = .03 and interaction between target visibility and consistency was not significant, *F* (2, 42) = 0.51, *p* = .56, *η*_p_^2^ = .02.

#### 3.2.2 Eye movements

Eye velocity signals were derived from position signals using a central difference algorithm on a ±10 ms interval. The previous research findings about smooth pursuit system have shown that smooth pursuit is more efficient in the horizontal than in the vertical dimension from newborns, infants, children, adolescent to adults (Engel et al., 1999; González et al., 2019; Grönqvist et al., 2006; Robert et al., 2014; Rottach et al., 1996; Vinuela‐Navarro et al., 2019). The horizontal–vertical tracking asymmetry is especially evident when subjects pursued a target moving on a circular trajectory (Collewijn & Tamminga, 1984; Grönqvist et al., 2006; Robert et al., 2014; Rottach et al., 1996). Based on all these studies, we only analyzed the horizontal component of eye velocity.

During visual tracking of a moving target, smooth pursuit response is usually separated into an open‐loop phase (the first 100 ms after initiation), and a closed‐loop or steady‐state phase (Lisberger et al., 1987; Tychsen & Lisberger, 1986). Pursuit in the open‐loop phase is primarily driven by the target's retinal image velocity, because an internal signal about the eye velocity is not yet available to the system. As eye velocity is gradually adjusted to target velocity, pursuit tends to be steady‐state and is mainly maintained by extraretinal inputs, such as efference copy (“eye velocity memory”), remembered target motion (“target velocity memory”) and object‐background consistency (Bennett & Barnes, 2004). In order to explore how extraretinal signals work to maintain a stable response with high gain, the eye velocity of horizontal smooth pursuit was plotted as a function of time.

The method of data analysis in studies of event‐related brain potentials (ERPs) as the reference, the horizontal smooth pursuit trace was segmented into 24 time intervals, with each interval lasting for 250 ms (Blair & Karniski, 1993). Twenty‐four mean velocity values were calculated on 22 subjects under each of three conditions. For each time interval, the statistical differences among the three consistency conditions were investigated by means of univariate analysis. Results of univariate analysis are exhibited in Table 5 and revealed that differences mainly occur at three time intervals: 100–250 ms, 2750–3000 ms, and 5750–6000 ms, consistent with the trend observed in the smooth eye velocity trace (Figure 4, 4). To determine whether there was any effect of each combination of independent variables on pursuit, we computed the velocity gain, which was defined as the ratio of the average eye speed to the target speed in a given time interval.

**TABLE 5:** Results of univariate analysis for each time interval

	*Df*_1_	*Df*_2_	*F*	*p*
1–250 ms	2	63	75.98	.000
215–500 ms	2	63	0.98	.412
501–750 ms	2	63	0.12	.988
751–1000 ms	2	63	0.14	.869
1001–1250 ms	2	63	0.07	.932
1251–1500 ms	2	63	0.53	.604
1501–1750 ms	2	63	4.20	.051
1751–2000 ms	2	63	1.37	.304
2001–2250 ms	2	63	0.00	1.00
2251–2500 ms	2	63	1.96	.196
2501–2750 ms	2	63	3.93	.059
2751–3000 ms	2	63	28.43	.000
3001–3250 ms	2	63	1.60	.255
3251–3500 ms	2	63	1.12	.369
3501–3750 ms	2	63	0.01	.993
3751–4000 ms	2	63	0.01	1.000
4001–4250 ms	2	63	0.45	.654
4251–4500 ms	2	63	0.15	.867
4501–4750 ms	2	63	1.84	.213
4751–5000 ms	2	63	4.08	.055
5001–5250 ms	2	63	0.55	.595
5251–5500 ms	2	63	0.14	.868
5501–5750 ms	2	63	0.23	.801
5751–6000 ms	2	63	17.53	.000

**FIGURE 4:**
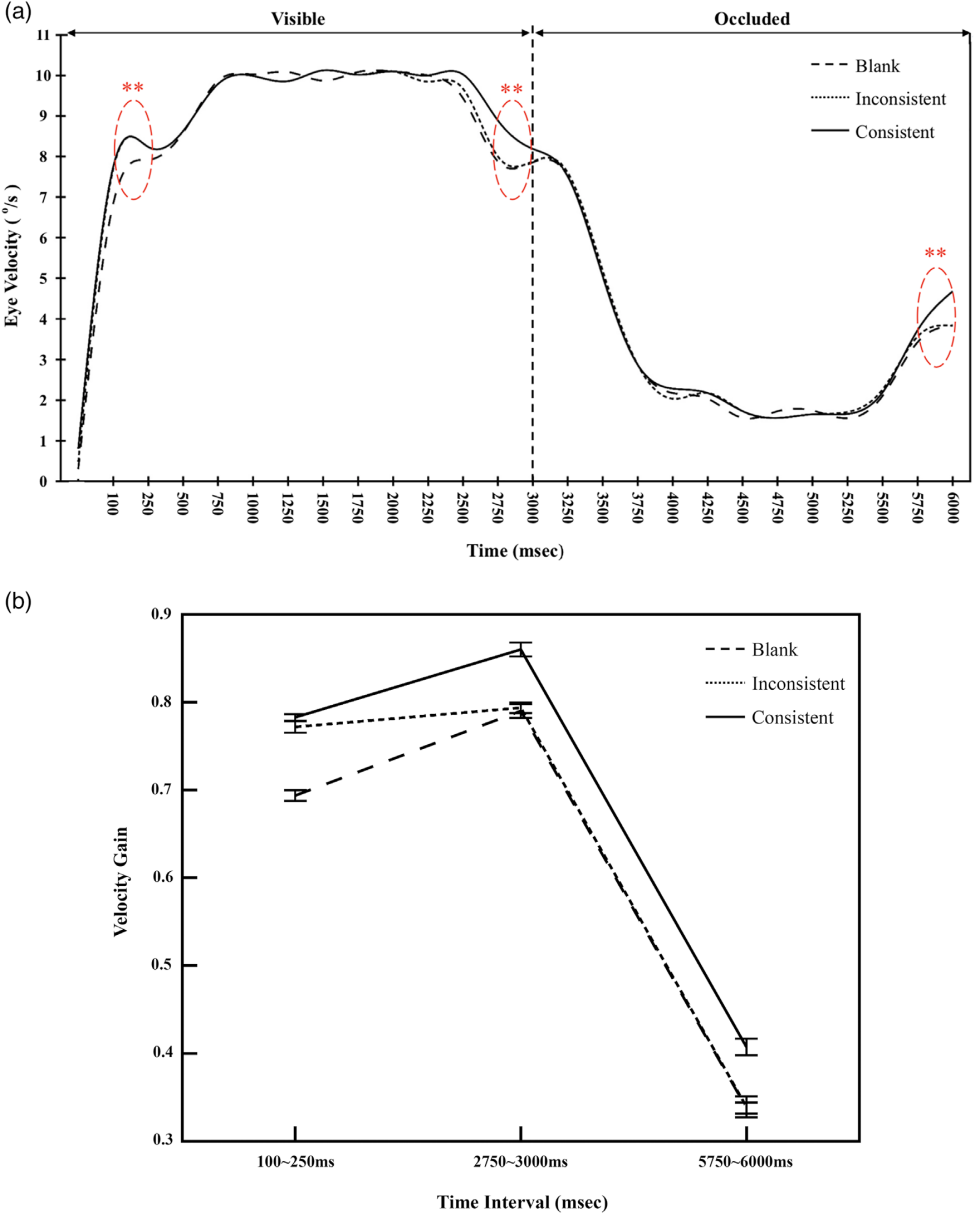
(a) Average velocity trace from all participants in prediction motion tasks. Saccades have been removed from the smooth eye velocity trace. (b) Velocity gain at three time intervals: 100–250, 2750–3000, and 5750–6000 ms. Eye movement velocity gain was defined as the ratio of the average velocity of the recorded eye movement to the target speed in a given time interval. (c) Representative example of eye velocity trace across trials from subject 10

**FIGURE 4:**
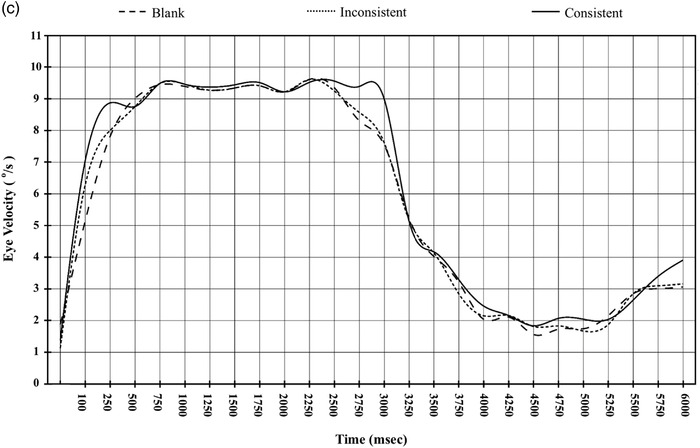
Continued

ANOVA was performed on the horizontal velocity gain, with the time interval (100–250 ms vs. 2750–3000 ms vs. 5750–6000 ms) and the scene consistency (blank vs. inconsistent vs. consistent) as two within‐participant factors. The results demonstrated a significant main effect of time interval, *F* (2, 42) = 3914.68, *p* < .001, *η*_p_^2^ = .99, suggesting that the mean gains varied as a function of the time interval. The main effect of the scene consistency was significant, *F* (2, 42) = 90.88, *p* < .001, *η*_p_^2^ = .81, and the time interval × scene consistency interaction was also significant, *F* (4, 84) = 8.44, *p* < .001, *η*_p_^2^ = .29.

Separate ANOVAs were performed for the three time intervals: 100–250, 2750–3000, and 5750–6000 ms. As can be seen in Figure 4b, during 100–250 ms, the main effect of scene consistency was significant, *F* (2, 42) = 92.34, *p* < .001, *η*_p_^2^ = .82, with gains higher in the trials with consistent backgrounds (*M* = 0.78, *SD* = 0.004) and inconsistent backgrounds (*M* = 0.77, *SD* = 0.007) than in the trials with blank backgrounds (*M* = 0.69, *SD* = 0.006), with 95% CI 0.07–0.11, *p* < .001 and 95% CI 0.06–0.10, *p* < .001, respectively. However, the difference in velocity gains between the inconsistent background and the consistent background conditions was not significant, *p *> .1. Furthermore, during 2750–3000 ms, there was also a significant main effect of scene consistency, *F* (2, 42) = 26.63, *p* < .001, *η*_p_^2^ = .56. Pairwise comparisons using Bonferroni correction revealed that the velocity gains were higher in the trials with consistent backgrounds (*M* = 0.86, *SD* = 0.008) than in the trials with inconsistent backgrounds (M = 0.79, *SD* = 0.006) and blank backgrounds (*M* = 0.79, *SD* = 0.008), with 95% CI 0.035–0.097, *p* < .001 and 95% CI 0.042–0.098, *p* < .001, respectively, but the means did not differ between the inconsistent background and the blank background trials, *p* > .1. Similarly, during 5750–6000 ms, there was also a significant main effect of scene consistency, *F* (2, 42) = 18.06, *p* < .001, *η*_p_^2^ = .46. Further comparisons using Bonferroni correction revealed that the velocity gains were higher in the trials with consistent backgrounds (*M* = 0.41, *SD* = 0.009) than in the trials with inconsistent backgrounds (*M* = 0.34, *SD* = 0.012) and blank backgrounds (*M *= 0.34, *SD* = 0.006), with 95% CI 0.032–0.104 and 95% CI 0.036–0.103, *p*s < .001, but the means did not differ between the inconsistent background and the blank background trials, *p* > .1. This is not surprising since the consistent background enhanced the tracking performance of the target circle under both visible‐ and memory‐guided tracking conditions.

## 4 DISCUSSIONS

A visual scene typically involves a set of hierarchically organized features, from local to global ones. While the majority of previous studies have demonstrated that Gestalt principles of grouping facilitate visual perception, the present study focuses on the contribution of global–local consistency to TTC estimation and oculomotor response in the PMT. Specifically, our results show that both observers’ behavioral response and eye movements were more accurate for a moving target in the consistent background than the blank background. Moreover, the consistency effect was found to be significant during both visually guided tracking and memory‐guided tracking. However, what differs from most studies is that facilitation of the inconsistent background on a moving object was unexpectedly found at early stage of visually guided tracking.

Navon (1977) argued that global processing is a necessary stage of perception prior to more fine‐grained analysis. The “global precedence effect” refers to these findings: (i) responses were faster to the global than the local level and (ii) when the levels were inconsistent, information at the global level interfered with (slowed down) responses to the local level, but not the other way around (Gerlach & Poirel, 2020). Global–local consistency effects could be generalized to visual search and recognition tasks (Aivar et al., 2014; Beanland et al., 2016; Castelhano & Pereira, 2018; Truman & Mudrik, 2018). Indeed, perception is temporally ordered so that global information is abstracted first and more local analysis is carried out some time later (May et al., 1995). Consequently, at early stage (100–250 ms) of pursuit tracking, tracking a target circle embedded in a circular background, the constant error between the actual and estimated TTC decreased, compared with a target circle in isolation. Moreover, significant differences occurred in horizontal velocity gain between the circular and blank background trials.

A smooth pursuit eye movement is induced when we look at a moving object to stabilize the image on or near the fovea. It is generally known that the first 100 ms of pursuit tracking are defined as an open‐loop response that occurs before the time of a feedback signal. In the early phase of initiation (0–20 ms), the eye starts to accelerate in the direction of the target, and in the later phase of initiation (20–100 ms), eye velocity is gradually adjusted to target velocity. Smooth pursuit initiation is driven by the target's retinal image velocity, because an internal signal about the eye velocity is not yet available to the system (Newsome et al., 1985; Dürsteler & Wurtz, 1988). As eye velocity is gradually adjusted to target velocity, however, pursuit tends to be steady‐state and is mainly maintained by extraretinal inputs (Bennett & Barnes, 2004). Generally, during steady‐state pursuit of an uninterrupted visible target, retinal, and extraretinal input cooperate to maintain a steady reaction with a high gain (Bennett & Barnes, 2003; 2004). Retinal input is obtained from the immediate feedback of visual motion signals, including image velocity and acceleration, while extraretinal input is driven by visual short‐term memory (VSTM), such as efference copy (“eye velocity memory”), remembered target motion (“target velocity memory”), volition, attention, and expectation (Bennett & Barnes, 2003; 2004; Barnes & Collins, 2008). At early stages of visual tracking, rapid global framework extraction provides the priority of the consistent local elements. Visual short‐term memory (VSTM) allows us to temporarily store and process global–local relevant information from the visual world. Hence, at later stage (2750–3000 ms) of visual tracking, differences in behavioral response and horizontal velocity gain between the circular and blank background trials are also significant.

Throughout visual tracking of a moving target, what often happens is that a moving target is momentarily obstructed by additional objects and vanished from view (Albright & Stoner, 2002). How do people react at about the correct time during PMT? Two classes of cognitive operations allow researchers to answer this question; DeLucia and Liddell called them cognitive clocking and cognitive motion extrapolation (DeLucia & Liddell, 1998). Based on the clocking strategy, participants approximate the time‐to‐contact (TTC) prior to occlusion onset with optic invariants (the Tau hypothesis; Lee, 1976). In particular, participants may calculate TTC from the ratio of exposed distance to hidden distance and the length of the object's visible motion; the clock would count the latter duration and the obstructed time (Rosenbaum, 1975). Conversely, the motion extrapolation strategy states that individuals create an inner cognitive representation of the object's visible movement and use this to determine the object's movement after it vanishes and to estimate TTC. Participants watch the target with spatial attention or pursuit eye movements, and then react when the gaze or spatial attention gets to the end of the obstruction.

Results provide the support of cognitive motion extrapolation depending on the pursuit system (DeLucia et al., 1998; Makin & Bertamini, 2014; Makin & Chauhan, 2014; Makin & Poliakoff, 2011; Makin et al., 2008; 2009). When a moving target is temporarily obstructed from view by other objects and there are no visual feedback signals, smooth pursuit eye velocity first diminishes substantially but it is maintained at a lowered gain because of extraretinal input. Extraretinal input is consisted of cognitive factors, including an expectation that the target will appear again later along its trajectory, and an inner cognitive representation of global–local consistency (Bennett & Barnes, 2003; 2004; Barnes & Collins, 2008; Spering & Gegenfurtner, 2008). These factors are utilized to extrapolate the object's motion and estimate TTC after it disappears. Results show that all participants’ CE, VE, and predictive smooth pursuit were worse in the occluded phase than in the visible phase, but the tracking accuracy for a target circle in circular backgrounds remained better than in blank contexts. The global–local consistency temporarily stored in VSTM allows us to extrapolate the target's position when observers predict the target circle reaching the end point (5750–6000 ms). The results are consistent with previous studies and suggest that the global–local consistency as a pivotal information source of extraretinal inputs constrains what to expect and where to look.

Another new finding of this study is that at early stage (100–250 ms) of visual tracking, horizontal velocity gains were higher for a moving target in circular background than in blank background trials, as well as for a moving target in triangular background than in blank background trials. Consequently, no differences were found between the circular and triangular background conditions. The facilitation of the inconsistent background on a moving object was not found during memory‐guided tracking and at later stage of visual tracking, but was found only at early stage of visually guided tracking. Whereas consistency advantages occur during both visually guided tracking and memory‐guided tracking. In the present experiment, both the triangular background and circular background moved in the same velocity and in the same rotating trajectory. Meanwhile, the spatial layout between a target and other distractors were invariant over time. This invariant structure can be available to capture attention. However, compared with the triangular background, the circular background allows observers to perceive the wheel‐like motion and then predict that the target to be tracked is likely to be a point on the rim of a rolling wheel (Steinbach, 1976).

## 5 CONCLUSIONS

Global–local consistency may act as an important information source to TTC estimation and oculomotor response in PMT. During closed‐loop phase of visual pursuit, global–local consistency could be activated within the first few hundred milliseconds to prioritize the deployment of attention and eye movement to component target. Meanwhile, it also removes ambiguity from motion tracking and TTC estimation under some unpredictable conditions, leading to the consistency advantage during smooth‐pursuit termination phase. In summary, the current study reveals that a coherent, consistent background can facilitate smooth‐pursuit initiation, steady state pursuit and smooth‐pursuit termination of a component object.

## CONFLICT OF INTEREST

The authors declare that they do not have any conflict of interests regarding the publication of this paper.

## AUTHOR CONTRIBUTIONS

Tingting Chen, Jinhong Ding, and Changhao Jiang developed the study concept. All authors contributed to the study design. Tingting Chen and Jinhong Ding collected the data and carried out the analyses. Tingting Chen and Changhao Jiang drafted the manuscript, and Jie Li, Haoqiang Liu, and Guang H. Yue provided critical revisions. All authors approved the final version of the manuscript for submission.

### PEER REVIEW

The peer review history for this article is available at https://publons.com/publon/10.1002/brb3.2444

## Data Availability

All data included in this study are available upon request by contact with the corresponding author.
